# Pectoralis Plane Block for Pacemaker Insertion: A Successful Primary Anesthetic

**DOI:** 10.3389/fsurg.2019.00064

**Published:** 2019-11-20

**Authors:** Ana C. Mavarez, Caroline I. Ripat, Maria R. Suarez

**Affiliations:** Department of Anesthesiology, Perioperative Medicine and Pain Management, Miller School of Medicine, University of Miami, Miami, FL, United States

**Keywords:** pectoralis block, pacemaker, regional anesthesia, analgesia, ultrasound-guided

## Abstract

Effective anesthesia, analgesia, and hemodynamic stability is important to maintain during pacemaker implantation surgery, especially in the elderly population and patients with compromised cardiac function. As a strategy to avoid the need for intravenous (IV) anesthetics, peripheral nerve block techniques may be used in these specific cases. We report a case of successful pacemaker implantation surgery in a patient with severe Aortic Stenosis (AS) and Sick Sinus Syndrome (SSS) using unilateral pectoralis plane block for surgical anesthesia. Since general anesthesia was considered risky, monitored anesthesia care utilizing peripheral nerve block was planned. A single shot left side pectoralis plane block (PECS II) was done under ultrasound guidance injecting a total of 20 mL of 0.5% Ropivacaine with 1% Lidocaine. No sedation was needed. The patient tolerated the procedure with no significant hemodynamic changes. Patient did not require opioids post-operative and was discharged home in stable condition the next day. This case highlights that PECS block can also provide effective surgical anesthesia for relatively long procedures avoiding the risk of complications associated with IV anesthesia in high risk cardiovascular patients. Additionally, these blocks can provide an opioid sparing option for post-operative management in pacemaker implantation surgeries.

## Background

Regional anesthesia carries less morbidity and mortality in the perioperative period than general anesthesia or Monitored Anesthesia Care (MAC) with sedation in the elderly population and patients with compromised cardiac function. Effective anesthesia and hemodynamic stability are important to maintain during pacemaker implantation surgery. Additionally, adequate post-operative analgesia for cardiac procedures helps in early recovery, ambulation and reducing post-operative complications (1). As a strategy for avoiding intravenous (IV) anesthetics, peripheral nerve block techniques may be used in these specific cases. We report a case of successful pacemaker implantation in a patient with Severe Aortic Stenosis (SAS) and Sick Sinus Syndrome (SSS) using unilateral pectoralis plane block I and II for surgical anesthesia and analgesia. Relevant anatomy for this regional anesthetic technique and benefits of this anesthetic technique compared to IV anesthesia are reviewed and discussed.

### Case Presentation

An 87-year-old woman (height 147 cm; weight 55 kg) with American Society of Anesthesiology physical status III, history of hypertension, SAS, Parkinson's disease, hyperlipidemia, gastrointestinal reflux disease, and limited functional capacity with <4 Metabolic Equivalents (METs) was admitted after recurrent syncopal episodes due to SSS and scheduled for pacemaker implantation. Pre-operative physical examination revealed hemodynamic stability with systolic ejection murmur in second right intercostal space, and limited range of neck motion, thyromental distance <3 fingerbreadths, mouth opening <2 fingerbreadths, edentulous with a Mallampati score of IV. Electrocardiogram showed sinus bradycardia with rhythm 50 beats per minute, and echocardiogram demonstrated mild concentric left ventricular hypertrophy, ejection fraction 60–65%, aortic valve area 0.4 cm^2^, and mild aortic regurgitation.

Patient was considered high risk for general anesthesia and so MAC utilizing peripheral nerve block was planned. Pre-procedure vitals were blood pressure 170/60 mmHg, heart rate 72 beats/min, respiratory rate 17 breaths/min, temperature 97.3 F, SpO2 96%. After placement of American Society of Anesthesiology standard monitors and supplemental oxygen, sterile technique and infiltration of skin and subcutaneous tissue with 1% lidocaine, a single-shot left side pectoralis plane block (PECS II) was performed under ultrasound guidance with Sonosite HF138 system and high-frequency linear transducer with an in-plane approach. The probe was placed in the infraclavicular region to identify axillary vasculature then moved caudally and laterally toward the 4th rib. A 22-gauge 50-mm stimulating 30 degree bevel Braun needle (Stimuplex A; Braun Medical) was inserted in the plane from medial to lateral direction and after gentle negative blood aspiration, 5 mL of 0.5% Ropivacaine with 1% Lidocaine was injected between the fascial plane of the pectoralis major and minor muscle, and 15 mL was injected in the fascial plane between the pectoralis minor and serratus anterior muscle. The local anesthetic was observed to separate the muscles and spread throughout the planes. No complications were observed.

Subcutaneous infiltration of 5 mL 1% Lidocaine was injected by the surgeon for skin incision. No IV sedation was needed throughout the surgery. The patient maintained a patent airway with oxygen supplementation via nasal cannula at 2 L/min, tolerating the procedure well, with no pain or restlessness during the 3-h surgery. Patient had no significant hemodynamic changes. Patient went to the recovery room following the procedure, then returned to the inpatient floor. She reported minimal pain for 24 h post-operatively (Visual analog scale scores ranging from 0 to 2) requiring no opioid analgesics. Patient was discharged home in stable condition on post-operative day one.

## Discussion

We report the successful use of ultrasound-guided PECS II block for pacemaker insertion in a patient with SAS and SSS. When these patients are scheduled for emergent non-cardiac surgery, this poses several hemodynamic challenges for the anesthesiologist. Neuraxial anesthesia is traditionally regarded as contraindicated in patients with SAS due to its sympatholytic effect, potentially causing loss of vascular tone and ultimately diminished cardiac output (2). Since patients with SAS have limited stroke volume, any major reduction in systemic vascular resistance may result in a sudden drop in perfusion pressure; therefore, induction and maintenance of general anesthesia in a patient with SAS should be done very carefully. Although pacemaker implantation is usually performed under general anesthesia or MAC sedation, in patients with compromised cardiac function, as in our patient with concomitant SAS, there exists a risk of significant mobility and mortality. There is no consensus on which anesthetic technique is safer for non-cardiac surgery in SAS patients. For this reason, PECS block as a relatively simple fascial plane infiltration technique can be used as it is associated with a good safety profile.

Regional anesthesia of the thoracic wall can be accomplished with various techniques including epidural, paravertebral block, intercostal nerve block, and PECS block. Due to the complications, such as inadvertent vascular puncture, epidural or intrathecal spread, pleural puncture, and pneumothorax, which can occur with the other techniques, PECS blocks have become more popular in the recent years.

The PECS block was introduced by Blanco as an alternative to paravertebral and epidural blockade to provide analgesia after breast expanders or subpectoral prosthesis used in reconstructive breast cancer surgery. The block was inspired by the infraclavicular block approach and the transversus abdominis plane block. The aim of the PECS block is to deposit local anesthetic into the interfacial plane between pectoralis major and minor muscles, targeting the lateral and median pectoral nerves (3). A second version of the PECS block was described later by the same author, the modified PECS block or PECS II. The aim of this approach was to provide local analgesia for the pectoral nerves and additionally the intercostobrachial, intercostals III-IV-V-VI and the long thoracic nerve. These nerves must be blocked to provide complete analgesia during breast surgery (4).

**Figure 1:**
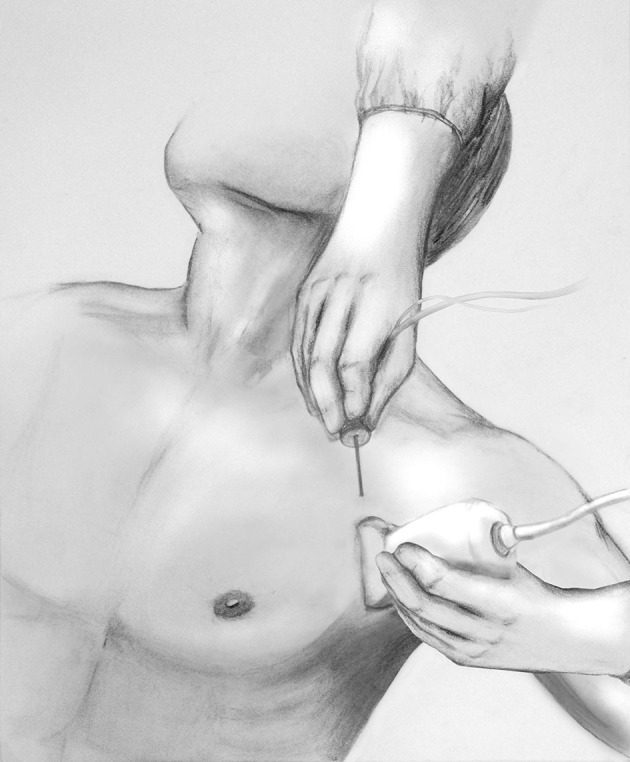
Transducer position and needle insertion point for PECS I and II block.

**Figure 2:**
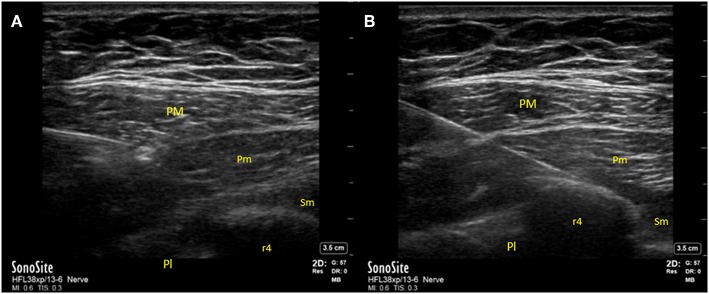
Ultrasound image with identification of relevant structures for Pectoralis plane block. **(A)** PECS I block and **(B)** PECS II block. PM, pectoralis major muscle; Pm, pectoralis minor muscle; Sm, Serratus anterior muscle; r4, fourth rib; Pl, pleura.

Ultrasound-guided technique of the PECS block is performed with the patient in supine position with the ipsilateral arm abducted 90-degrees. A high-frequency linear ultrasound probe is placed at the mid-infraclavicular level and angled inferolaterally (Figure 1) with localization of the axillary artery and vein and subsequent identification of the pectoralis minor and serratus anterior muscles. The 2nd rib is located immediately under the axillary artery, and the probe is moved caudally and laterally to count the 3rd and 4th rib. At the level of the 4th rib, after skin infiltration with lidocaine, the needle is inserted in-plane from medial to lateral direction, in an oblique manner to ensure the location of the needle tip in the fascial planes between the muscles. Administration of local anesthetic between the fascial plane of the pectoralis major and minor muscle targets the lateral and medial pectoral nerve (PEC I) and between the pectoralis minor and serratus anterior muscle it targets the thoracodorsal, intercostobrachial, intercostals III to VI and the long thoracic nerve (PECS II). When performing PECS II block, PECS I is implicit (Figure 2).

Traditionally, PECS block has been used exclusively for breast surgery to provide post-operative pain management or as part of multimodal analgesia to reduce opioid consumption. Relevant case reports include PECS block in combination with an intercostal nerve block with IV dexmedetomidine for the sub-pectoral implantation of a cardiac resynchronization therapy device (5), PECS block for implantable cardioverter defibrillator insertion in young patients with Duchenne muscular dystrophy with intercostal block and IV sedation (6), PECS for ICD in a super obese patient (7), and relocation of an infected cardiac pacemaker generator under PECS II block using IV midazolam (8).

In our case report, PECS II block was used alone as the primary anesthetic technique without using any sedation or IV anesthetics. PECS II block was also used in this case for perioperative analgesia, as patient did not receive any opioids pain medications within 24 h after the procedure and was discharged home comfortable and hemodynamically stable.

In summary, PECS block has been described in the setting of breast surgery to provide chest wall post-operative analgesia (3, 4) but our findings suggest that PECS block can also provide effective surgical anesthesia as the primary anesthetic technique for relatively long procedures avoiding the risk of complications associated with IV anesthesia for pacemaker implantation surgery in high risk patients.

## Ethics Statement

Institutional Review Board approval is not required for case reports at the University of Miami Hospital. Written informed consent was obtained from the individual(s) for the publication of any potentially identifiable images or data included in this article.

## Informed Consent

Written consent was obtained from the patient daughter before submission of this report. Patient has cognitive and intellectual impairment.

## Prior Presentation

This case was presented at the 2019 44th Annual Regional Anesthesiology and Acute Pain Medicine Meeting of the American Society of Regional Anesthesia and Pain Medicine in Las Vegas, April 11 to 13, 2019.

## Author Contributions

AM helped with literature review, drafting, writing the manuscript, and provided the illustration. CR helped drafting and editing the manuscript. MS helped editing the manuscript and provided the ultrasound images.

### Conflict of Interest

The authors declare that the research was conducted in the absence of any commercial or financial relationships that could be construed as a potential conflict of interest.
